# Dynamics of the thalamo-cortical system driven by pulsed sensory stimulation

**DOI:** 10.1186/1471-2202-14-S1-P67

**Published:** 2013-07-08

**Authors:** Arne Weigenand, Michael Schellenberger Costa, Hong-Viet Ngo, Lisa Marshall, Thomas Martinetz, Jens Christian Claussen

**Affiliations:** 1 Institute for Neuro- and Bioinformatics, University of Lübeck, 23562 Lübeck, Germany; 2 Graduate School for Computing in Medicine and Life Sciences, University of Lübeck, 23562 Lübeck, Germany; 3 Department of Neuroendocrinology, University of Lübeck, 23562 Lübeck, Germany; 4 Institute of Medical Psychology and Behavioral Neurobiology, University of Tübingen, 72074 Tübingen, Germany

## 

There exists a large body of evidence pointing to an essential role of sleep in memory consolidation [1-3]. In particular non-REM sleep seems to be important for consolidating declarative memories [4]. Boosting the so-called slow oscillations (<1 Hz) during non-REM sleep via transcranial electric stimulation leads to a potentiation of memory [5]. It has also been demonstrated that slow oscillations can be induced by optogenetic, magnetic and acoustic stimulation [6-8]. Here we present data from human sleep studies and modeling results on the thalamo-cortical system under sensory stimulation, that give new clues for effective stimulation protocols. We use a population model that exhibits important features of brain activity during non-REM sleep, e.g. spindles, cortical

slow oscillations with gamma activity and clock-like delta oscillations. The model aims at reproducing evoked responses of auditory and visual stimuli at several frequencies. We extend previous results on the phase-dependent response of isolated cortex [9] to stimuli which are time-locked to spindle and slow oscillation events and test the hypothesis that the main factor determining thalamic gating properties in non-REM sleep is the phase of the cortical slow oscillation.
